# 3D printed objects do not impact the behavior of a coral-associated damselfish or survival of a settling stony coral

**DOI:** 10.1371/journal.pone.0221157

**Published:** 2019-08-16

**Authors:** Emily J. Ruhl, Danielle L. Dixson

**Affiliations:** School of Marine Science and Policy, University of Delaware, Lewes, DE, United States of America; Department of Agriculture, AUSTRALIA

## Abstract

3D printing technology offers significant advantages in the development of objects and tools across an array of fields and has been implemented in an increasing number of ecological studies. As rates of degradation or chemical leaching of 3D printed models has not been well documented under environmental conditions, it is essential to examine if these objects will alter the behavior or impact the survivorship of the focal species prior to widespread implementation. Here, we explored the efficacy of using 3D printed models in coral reef behavioral research, an area of study where this form of additive manufacturing could offer significant advantages. Coral-associated blue-green chromis (*Chromis viridis*) individuals were exposed to natural and 3D printed coral habitats, and larval mustard hill coral (*Porites astreoides*) were offered 3D printed substrate as a settlement surface. Habitat association and behavioral analyses indicated that *C*. *viridis* did not discriminate or display modified behaviors between 3D printed and natural coral skeletons or between 3D printed materials. *P*. *astreoides* displayed significantly higher settlement when provided with 3D printed settlement surfaces than when provided with no settlement surface and settled at similar rates between 3D printed surfaces of differing materials. Additionally, growth and mortality of *P*. *astreoides* settled on different 3D printed surfaces did not significantly differ. Our results suggest that the 3D printed models used in this study are not inherently harmful to a coral reef fish or species of brooding coral, supporting further exploration of the benefits that these objects and others produced with additive manufacturing may offer as ecological research tools.

## Introduction

3D printing is a form of additive manufacturing technology that fabricates physical objects from model data created by laser-scanning an object, photogrammetry, or computer-aided design software. During the printing process, models are digitally sliced into 2D layers, then built from the base upwards through the deposition of melted material [1]. Technological advances in 3D printing have made this process applicable and valuable in developing objects and tools across an array of fields. For instance, 3D printed implants [2] and prosthetics [3] are designed specific to individual medical needs, buildings are constructed using industrial-sized printers [4], and 3D printed objects are used as tangible educational tools [5].

Ecologists have also recognized the advantages of 3D printing to rapidly create small-scale, precise replicates of focal species, stimuli, or habitats for research and management purposes. For example, decoy emerald ash borers (*Agrilus planipennis*) were used to bait traps within a heavily infested North American ash plantation, eliminating the need to obtain live specimens for trap deployment [6]. 3D printing was also used to mimic naturally varied egg shapes and sizes of the parasitic brown-headed cowbird (*Molothrus ater*) to identify the cause of host-egg rejection by the American robin (*Turdus migratorious*) without sacrificing genuine eggs [7]. Other behavioral studies have used 3D models to understand the visual effect of floral traits on pollinators [8] and predation rates on lizards [9].

3D printing has also afforded new methods to study behaviors and habitat preferences of aquatic species, which often occupy habitats that are inherently challenging to study or replicate. 3D printed experimental habitats can act as structural replicates or specifically manipulated alternatives of natural habitats, providing a more accurate understanding of the properties of structure that mediate ecological interactions. For instance, the interstitial space of a 3D printed oyster reef was quantitatively manipulated to understand the structural properties that mediate foraging success of the blue crab (*Callinectes sapidus*) [10]. As individual oysters grow irregularly, the use of 3D printed shells allowed for consistency and repeatability of the experimental habitat.

Utilizing 3D printed models could benefit research conducted in other structurally complex and vulnerable systems, such as coral reefs. Coral reef systems are in global decline due to a combination of anthropogenic stressors [11–12], yet customary research methods may require manipulation, destruction, or interference with what little habitat remains to understand the complex processes driving coral reef ecosystems. Thus, the application of artificial 3D printed corals could offer more quantitatively repeatable and less invasive alternatives to investigate the consequences of this habitat loss on reef organism behavior.

While 3D printing has the potential to advance the discipline of coral reef behavioral ecology, it is pertinent to assess the responses of species under controlled conditions to these artificial objects, frequently created with thermoplastics, prior to widespread implementation of 3D printed models *in situ*. Here, we explored the efficacy of using various 3D printed models and materials in coral reef research by investigating 1) the behavior responses of the blue-green chromis (*Chromis viridis*) when exposed to 3D printed and natural coral skeletons, and 2) the colonization and survivorship of Caribbean mustard hill coral (*Porites astreoides*) on 3D printed substrate.

## Methods

### 3D print technology use in fish behavior experiments

#### Study species

*C*. *viridis* is a coral-associated damselfish in the family Pomacentridae [13], making this fish an ideal focal species for habitat association experiments. *C*. *viridis* (n = 60, SL 4.1 ± 0.07 cm) were obtained from a local aquarium store (The Fish Bowl, Dover, DE) and held in a 40L glass aquarium connected to a 590L sump on a re-circulating system. Tank water was maintained at 32 ppt salinity, 8.1 pH, and 26°C. Overhead timed lighting was set at 14:10 light to dark ratio. Fish were fed pellets daily to satiation (Sustainable Aquatics hatchery diet pellets, Jefferson City, TN). This study was carried out in accordance with the protocols outlined by the Institutional Animal care and Use Committee. The protocol was approved by the Office of Laboratory Animal Medicine at the University of Delaware (AUP 1305).

#### 3D model creation and printing

Two species of scleractinian coral skeletons, *Acropora formosa* and *Pocillopora damicornis*, were used as models to create 3D printed corals. *P*. *damicornis* is a bushy coral, whereas *A*. *formosa* has long, wide set branches. Morphologically different species were chosen to determine if observed *C*. *viridis* behavior patterns were in response to the 3D printed filaments or visual cues of the structure. Both species significantly contribute to Pacific reef matrices, playing important roles in benthic communities [14] and serving as protective habitats for *C*. *viridis*.

**Fig 1:**
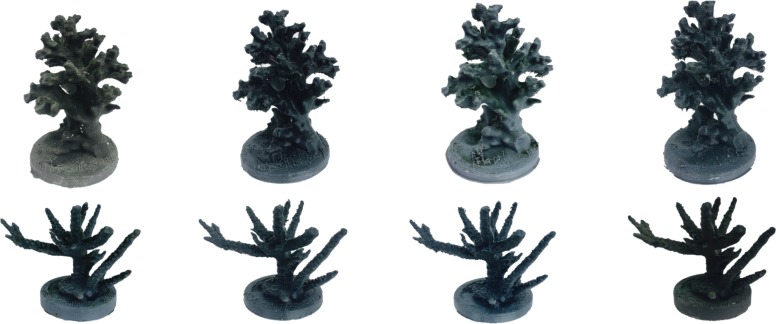
Replicates of *P*. *damicornis* (top) and *A*. *formosa* (bottom) control corals 3D printed with nGen, XT, PLA, and SS filament, respectively.

Each coral skeleton was photographed from 50 angles against a white background using an Apple iPhone 6s. Photographs were uploaded into a photogrammetry program (Autodesk ReMake) and stitched into a 3D model. Models were exported and printed using the slicing program Cura (21.08) and the Lulzbot Taz 5, Lulzbot Taz 6, and Makerbot Replicator 2 machines. All models were printed with the same dimensions as the natural control coral skeletons (10×7.2×7.8cm). Four filament treatments per coral species were used to determine if different print polymers would affect the suitability of 3D printed objects (Fig 1). Filaments chosen represented a range of those available and most feasible to use due to cost and weight. Two Colorfabb co-polyester based filaments were chosen for their durability (hereafter nGen and XT); a Colorfabb PLA/PHA filament was selected for its biodegradable property (hereafter PLA); and a Proto-Pasta PLA filament was chosen for its biodegradable property and the incorporation of stainless-steel shavings (hereafter SS). All 3D printed models were conditioned in seawater for one-week prior to use.

#### *C*. *viridis* behavior experiments

A cafeteria-style arrangement was used to determine preferences between the four filament options and a natural coral skeleton control [15]. All five coral treatments of a single species were arranged in a circular pattern spaced 50cm apart from the corals directly adjacent in a 1.8m diameter tank filled to 45cm. For each trial, coral species and treatment order were randomized. An individual fish was placed into an 18cm diameter mesh cylinder (1cm^2^) at the center of the experimental tank and left to habituate for 15-minutes (*A*. *formosa* n = 29, *P*. *damicornis* n = 15). The cylinder’s construction allowed the fish to observe all habitat treatments without having access to them. After the habituation period, the cylinder was slowly raised to begin the 15-minute observation period.

Habitat associations were recorded continuously. A fish was considered associated with a habitat if it was within 5cm of the habitat in either a stationary or slow swimming position, a metric previously used to assess damselfish habitat associations [16]. A second consecutive association at the same habitat was recorded if the fish swam more than 5cm from a coral and returned. Fish were unable to see the observer during trials. Each fish was used only once.

Association times at each coral treatment were transformed into the proportion of the total time associated with any coral during a given trial. The proportion of association time was first compared between the coral species (Welch’s t-test) and then between filament treatments (Kruskal-Wallis test with Bonferroni correction, Dunn’s test post-hoc comparisons).

Behaviors of individual fish were also observed for activity level while in a tank with a single coral treatment to determine if 3D printed habitats would compromise their ability to behave defensively and seek refuge [17]. To do so, a single coral treatment was placed in the back-left corner of an 6L observation tank. Tanks were allowed flow-through water (26°C) and aeration throughout trial periods. The tanks were covered on three sides to block visual cues from adjacent tanks. *C*. *viridis* were tested individually and allowed 24 hours to habituate.

Activity level was measured by the mean frequency and extent of an individual’s movement throughout the tank and in relation to the habitat. Observation tanks were divided into four equal vertical (4cm) and six equal horizontal areas (4cm) marked with lines. If the fish was within the coral habitat, the distance was marked as 0cm, and the length of time inside was recorded. The total number of lines crossed during the 10-minute observation period was recorded. At least half the body length must have crossed a line to be recorded as movement. Each fish was used only once (n = 6 filament treatment^-1^ coral species^-1^).

The difference in total counts of line crosses, time spent in shelter, and maximum distance ventured from shelter were individually compared between the coral species (Welch’s t-test), and then between all filament treatments (one-way MANOVA).

### 3D print technology use in coral settlement experiment

#### Study species

*P*. *astreoides* is common throughout the Caribbean and Atlantic basin, reproducing by brooding planulae larvae around the lunar cycle [18]. *P*. *astreoides* planulae larvae are hardy and commonly used in laboratory experiments. During the May 2017 lunar spawning event, 42 adult colonies were collected from Wonderland Reef, FL Keys (24° 34.130’ N, 81° 22.868’ W) using the spillover method [19]. Larvae were packaged in filtered seawater and shipped the day after the release to the University of Delaware for experimentation.

#### 3D model creation and printing

Using similar methods described for modeling the corals, a terracotta tile was used as a model to create 3D printed settlement tiles that provided textured substrate *P*. *astreoides* have been shown to prefer [20]. Tiles were 3D printed (4×4×0.2cm) using the same four filament treatments as in the fish behavior experiments (nGen, XT, PLA, and SS). All tiles were conditioned in seawater for one-week prior to use.

#### *P*. *astreoides* settlement experiment

Settlement of *P*. *astreoides* was tracked under controlled conditions in 500ml glass aquaria, filled with 200mL of artificial seawater that was changed every 48 hours. In each experimental aquarium, one 3D printed tile treatment was placed on the bottom, while control aquaria contained no tile (n = 12). As the intention of this experiment was only to compare settlement rates of *P*. *astreoides* across different 3D printed materials, no treatments containing non-3D printed materials were included. Aquaria were randomly arranged in a 380L water bath to ensure constant temperature (26°C) throughout the experiment.

Seven *P*. *astreoides* planulae larvae were pipetted into each aquarium, and settlement was assessed daily for 14 days. Settlement was scored as the number of planulae that had attached and metamorphosed on any surface. Settled spat were considered attached to a surface if they could not be dislodged by light agitation of the water. Spat that had metamorphosed but were still free-floating in the water column were scored as dead.

A comparison of the total spat that settled within each treatment aquaria to the total spat that settled within the control aquaria was conducted (chi-square analysis). It was assumed that the absence of a provided settlement surface within the control treatment did not deter planulae larvae from settling on the glass. In this analysis, settlement included *P*. *astreoides* that settled anywhere in the aquaria for all treatments (i.e. glass or tile) at any point during the settlement period. Coral larvae that did not settle or died before settling were not included in this analysis. To analyze total settlement between the 3D printed tile treatments, only *P*. *astreoides* that settled on each tile at any point during the settlement period were included (repeated measure ANOVA).

After the 14-day settlement period, only *P*. *astreoides* settled on the treatment tiles were tracked for growth for 12 weeks. Tiles were placed on elevated racks directly in the 380L water bath to provide a continual supply of aerated seawater during the growth period. A pipe cleaner was used weekly to keep tiles clean of algae to prevent additional factors from impacting coral growth. The sizes of the coral larvae were calculated by measuring the individual skeletal diameters every 3^rd^ week using an imaging microscope (AmScope 10X) and ImageJ software [21]. Surface area of each individual spat was then calculated from the skeletal diameters.

Growth rates were defined as the rate of change in surface area over time of settled individuals that survived the entire 12-week growth period (repeated measure ANOVA). Growth rate was used as the dependent variable to account for initial size differences of the individual corals. Percent mortality of *P*. *astreoides* between treatment conditions was analyzed by comparing the total number of *P*. *astreoides* that settled at any point on each tile treatment and the final count that survived at the end of the 12-week growth period (one-way ANOVA).

## Results

### *C*. *viridis* behavior experiments

For the cafeteria-style experiment, no significant difference was found between the total proportion of time that *C*. *viridis* associated with any of the filament treatments between the *A*. *formosa* and *P*. *damicornis* treatments (Welch’s t-test: t_(27.247)_ = 1.018, p = 0.32). The lack of difference in association times between the coral species indicated that *C*. *viridis* did not make habitat choices based on the morphology of the corals. Thus, the data for the two coral species treatments were combined.

**Fig 2:**
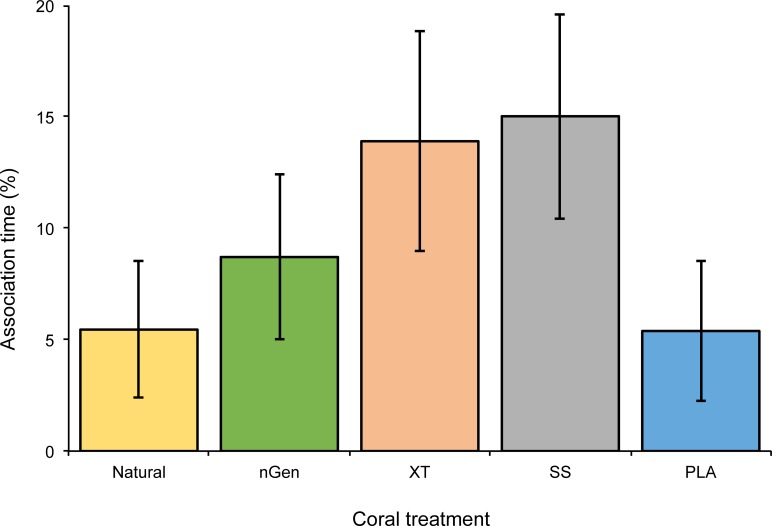
*C. viridis* spent in association with any of the coral habitat treatments (n = 44). Mean percent time (± SE).

*C*. *viridis* associated with any of the habitat treatments for 48% (± 6.7) of the time, which was not significantly different than the amount of time spent not associating (Welch’s t-test: t_(43)_ = -0.228, p = 0.82). When considering only association time, *C*. *viridis* were not significantly selective of any of the coral habitats (Kruskal-Wallis: H_(4)_ = 7.896, p = 0.1) (Fig 2).

For the activity level experiment, differences in behavioral responses were first analyzed between *C*. *viridis* exposed to either *A*. *formosa* or *P*. *damicornis* treatments. No significant differences were found for time spent in the habitat provided (Welch’s t-test: t_(58)_ = -0.81, p = 0.42), maximum distance ventured from the habitat (Welch’s t-test: t_(58)_ = 0.44, p = 0.66), modal distance ventured from the habitat (Welch’s t-test: t_(58)_ = 1.08, p = 0.28), or the number of lines crossed (Welch’s t-test: t_(44.16)_ = 0.59, p = 0.55) regardless of habitat treatment. Data for coral species treatments were again combined to assess responses to the 3D filaments.

**Fig 3:**
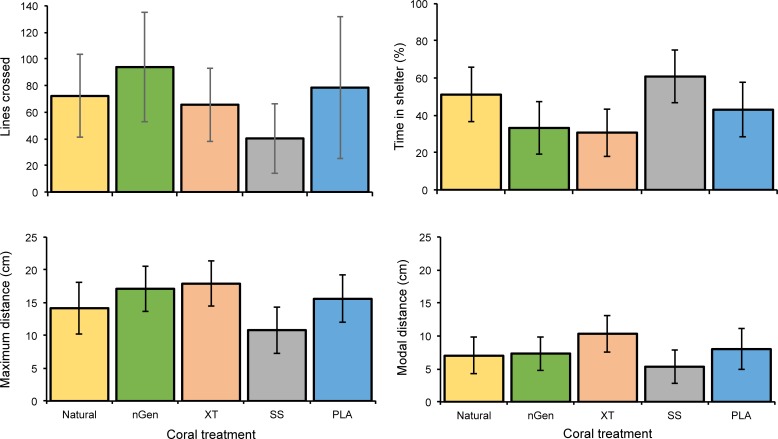
Mean behavioral responses (± SE) by *C*. *viridis* when exposed to 3D printed or coral skeleton habitats (n = 12).

No significant differences were found between the five treatments for any of the behavioral traits analyzed (MANOVA: Pillai’s trace = 0.19, F_(4, 55)_ = 0.70, p = 0.8) (Fig 3).

### *P*. *astreoides* settlement experiment

**Fig 4:**
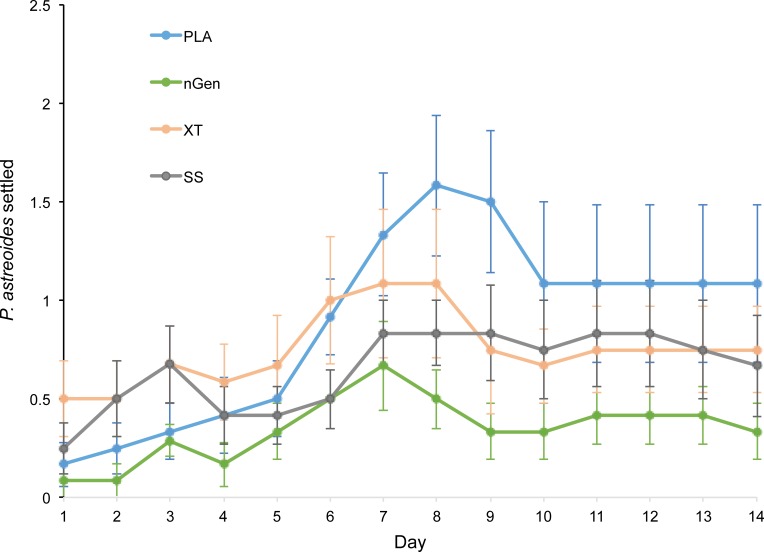
Mean number (± SE) of *P*. *astreoides* settled on each 3D printed treatment tile during a 14-day settlement period.

All 3D printed tile treatments were found to have significantly higher settlement compared to the control aquaria (Table 1). Total settlement on tiles did not differ significantly between the treatments (repeated measures ANOVA: F_(3, 668)_ = 1.355, p = 0.2557; Fig 4).

**Table 1:** All 3D printed tile treatments were found to have significantly higher settlement compared to the control aquaria.

Treatment	Total Settled	χ ^2^	df	p-value
Control	15	-	-	-
nGen	32	27.43	1	P<0.001
XT	52	88.04	1	P<0.001
SS	36	71.11	1	P<0.001
PLA	41	46.57	1	P<0.001

Percent post-settlement mortality of *P*. *astreoides* settled on each tile treatment did not differ significantly between the experimental treatments (one-way ANOVA: F_(3, 31)_ = 0.5943, p = 0.6235; Table 2).

**Table 2:** No significant differences in post-settlement mortality of *P*. *astreoides* settled on different 3D printed tiles were found.

Treatment	Settled on Tile	Tile Post-Settlement Mortality	Post-Settlement Mortality (%)	SE (%)
nGen	11	9	81.82	16.77
XT	17	9	52.94	13.06
SS	13	8	61.54	16.77
PLA	20	13	65.00	13.71

No significant differences in the average growth rates of *P*. *astreoides* on the different tile treatments (F_(3, 62)_ = 1.099, p = 0.357; Table 3) were found. This analysis included only settled individuals that survived the entire 12-week growth period.

**Table 3:** No significant differences were found in average growth rates of *P*. *astreoides* that survived a 12-week growth period on 3D printed tiles.

Treatment	Total Settled Survivors	Average Growth Rate (mm^2^week^-1^)	SE (mm^2^week^-1^)
nGen	2	0.078	0.01
XT	8	0.201	0.01
SS	5	0.211	0.02
PLA	7	0.162	0.05

## Discussion

Evaluating coral reef fish habitat preferences and behavior when exposed to various stressors has elucidated survival mechanisms of coral reef fishes, such as activity level or seeking refuge, and how those mechanisms may be impacted when in unfavorable habitats [22–23]. Despite not having live coral polyps, the natural coral skeletons used in this study should have acted as suitable refuges for *C*. *viridis* by providing protective structure [24]. By exposing *C*. *viridis* to various 3D printed corals simultaneously, designed to provide replicate protective structures, we were able to determine if these artificial habitats would act as a stressor.

When presented with the natural and artificial habitat treatments, *C*. *viridis* showed no preference, suggesting that none of the filaments used acted as a deterrent. When observing behavioral responses, *C*. *viridis* did not interact with any of the artificial habitats in a significantly deviant manner compared to a natural coral skeleton. As increased shelter use is a well-known defensive behavior in damselfish [25], the behavioral similarities observed between *C*. *viridis* exposed to artificial and natural coral indicated the 3D printed corals did not elicit weakened defensive behaviors.

Similarly, activity level of *C*. *viridis* was not significantly altered by the 3D printed corals. Activity level has been equated with a measure of a fish’s boldness that can indicate the likelihood of food acquisition, courting behavior, and encounter rates with predators [26]. Stimuli that affect patterns in activity levels of reef fishes could have significant interspecific and intraspecific impacts [27]. 3D printed corals were able to successfully replicate the target complex habitat without compromising *C*. *viridis’* activity patterns, suggesting the use of these models for similar research *in situ* is justifiable.

The successful recruitment of reef-building corals is a crucial process for ensuring the longevity of coral reef ecosystems. Once recruited, post-settlement facets of survivability such as predation, habitat suitability, and water quality affect the viability and growth rate of the coral spat [28]. Thus, it is important to understand how the survivorship of coral spat may be affected if they settle on 3D printed materials intended for use as settlement sites on natural reefs. Under controlled conditions, *P*. *astreoides* chose to settle significantly more in aquaria containing 3D printed tiles compared to control aquaria with no tiles. This result suggests the 3D printed tiles were successfully designed to provide a textured surface that recruiting corals have shown to prefer [29]. While it was not in the scope of the current study to broadly compare settlement rates on 3D printed tiles to more traditional settlement surfaces, this assessment would be valuable for exploring potential alternatives to conventional methodologies.

Settlement time for most treatments was comparable to settlement rates observed on more commonly used materials. In a similar experimental design, the settlement rate of *P*. *astreoides* on limestone tiles, pre-conditioned *in situ*, was 12.5% (± 4.95) after a one-week settlement period [30]. After one week in the current study, all treatments had a higher settlement rate on the 3D printed tiles. This is especially significant as the 3D printed treatment tiles were not pre-conditioned with a surface biofilm known to induce settlement and metamorphosis in recruiting corals [31]. However, the mean growth rates of spat on the 3D printed tile treatments were more than 50% lower than *P*. *astreoides* settled onto pre-conditioned limestone tiles. As spat growth rate has been shown to be inversely proportional to mortality risk [32], a slow growth rate could have significant implications for juvenile survivorship. Future experiments should consider pre-conditioning 3D printed tiles to differentiate how spat growth is impacted by the presence of a biofilm and the material.

Percent mortality of corals settled on treatment tiles was comparable to rates observed for *P*. *astreoides* in other studies. On conditioned limestone tiles, survival of *P*. *astreoides* spat settled under controlled conditions was 10% after 24 hours *in situ* [33]. After 12 weeks in the current study, survival ranged from 21% (nGen) to 48% (SS). While the rate of survival for *P*. *astreoides* settled on 3D printed tiles under controlled conditions is higher than the rates found in other studies, it is likely that mortality would increase if tracked *in situ*. Newly settled corals are extremely vulnerable to predation and abiotic stressors, resulting in low rates of juvenile survivorship [34]. However, it can be inferred from the results of this study that 3D printed materials did not have increased immediate or latent post-settlement effects on *P*. *astreoides* survival under controlled conditions. Future studies should track growth and survivorship of juvenile corals on 3D printed substrate in a reef setting.

Our findings indicate that exploring the use of 3D printed objects *in situ* should be benign in coral reef systems and could be appropriate for evaluating certain reef organism behaviors and habitat preferences. As coral reef ecosystems are highly dynamic environments [35–36], field studies are the next step to investigate the efficacy of using 3D printed objects to facilitate ecological research.

Reef fish predation [37], recruitment [38], and habitat choice [39] studies traditionally require construction of isolated patch reefs, where live coral is detached from the contiguous reef. Live coral may also be experimentally bleached to investigate interspecific effects of habitat degradation [40]; 3D printed corals could alleviate stress on the reef that would conventionally be incurred in similar experiments. For instance, 3D printed Caribbean staghorn corals (*Acropora cervicornis*), used to assess habitat preferences of reef fishes, were shown to attract similar numbers of fish as natural *A*. *cervicornis* when created with sufficient structural complexity [41]. Demonstrably, 3D printed models mimicking natural habitats can act as suitable proxies in controlled and dynamic environments and could be used in place of live coral when appropriate.

While coral settlement studies *in situ* are typically not disruptive to coral reef systems, 3D printed substrate could allow for novel methodologies in conducting this research. For instance, printed substrate could be designed to compare settlement rates, growth, and survival of different coral species across specific surface complexities or cryptic microhabitats [42]. This information could inform management practices by tailoring efforts to the needs of individual species.

3D printing offers a cost-effective, easy, and precise method for quickly replicating treatments or complex structures with irregular morphologies. These models can be designed for research with specific species in mind and can be created with biodegradable filaments, such as PLA, to alleviate the effects of adding harmful microplastics into the marine environment if models are lost or broken *in situ*. In a similar vein, the use of 3D printed objects in ecological research could lead to innovative conservation measures, such as complex 3D printed artificial reefs. There is still much to understand about the factors that influence the behavior and survival of coral reef organisms, and this study supports the consideration of a new, less invasive, and more tailored methodology for increasing that understanding.

## Supporting information

S1 Dataset Complete raw data set. (XLSX)
